# Steroids for the treatment of viral encephalitis: a systematic literature review and meta-analysis

**DOI:** 10.1007/s00415-023-11715-0

**Published:** 2023-04-15

**Authors:** Emira Hodzic, Rodrigo Hasbun, Alejandro Granillo, Anna R. Tröscher, Helga Wagner, Tim J. von Oertzen, Judith N. Wagner

**Affiliations:** 1 Institute of Applied Statistics, Johannes Kepler University, Linz, Austria; 2 Division of Infectious Diseases, Department of Internal Medicine, McGovern Medical School, UT Health, Houston, TX USA; 3 Department of Neurology, Evangelisches Klinikum Gelsenkirchen, Teaching Hospital University Duisburg-Essen, Munckelstrasse 27, 45879 Gelsenkirchen, Germany; 4 Johannes Kepler University, Linz, Austria

**Keywords:** Infectious diseases, Neurovirology, Steroids, Intensive care

## Abstract

**Background:**

Specific antiviral treatment is only available for a small subset of viral encephalitis (VE). Adjunctive steroids are used, but there is scant evidence evaluating its utility. We present a systematic review and meta-analysis on the outcome of steroid use in VE.

**Methods:**

We conducted a systematic literature review and reported it according to the Preferred Reporting Items for Systematic Reviews and Meta-Analyses (PRISMA) standards. Two observational studies from unpublished or partially published data were added. For the meta-analysis, we employed the metaphor package of the statistical software R-4.3.1.

**Results:**

We screened 378 studies and included 50. 155 patients were added from the Houston and Linz cohorts. Individual data were available for 281 persons, 120 (43%) of whom received steroids. The most common pathogens were herpes simplex virus 1, West Nile virus, and measles. Study designs and patient outcomes were heterogeneous. Only three of the trials report an advantage of steroid therapy. Steroid-induced side effects were scarce. Ten cohorts were included into the meta-analysis. For the pooled data, the null hypothesis could not be rejected (*p* = 0.245) using a random effects model, i.e., a benefit of steroid treatment on survival in VE could not be shown.

**Conclusions:**

Steroids as potent anti-inflammatory agents may act through a reduction of secondary inflammation-mediated damage. Our data do not support the use of steroids in VE. However, multiple shortcomings apply. Standardized controlled trials are needed to investigate optimal dosing and timing of steroid administration and to explore potential subgroups that could benefit.

**Supplementary Information:**

The online version contains supplementary material available at 10.1007/s00415-023-11715-0.

## Introduction

Encephalitis is an acute neurological syndrome characterized by altered mental status in combination with two or more secondary diagnostic criteria (fever, new epileptic seizures, or neurological deficits, cerebral spinal fluid pleocytosis, specific alterations detected by neuroimaging or electroencephalography) [1]. The cause is unknown in approximately half of all cases. In the remainder, up to 50% are due to viral pathogens [2]. While specific antiviral treatment is available for a small subset of viral encephalitides—most notably acyclovir for herpes simplex and varicella zoster virus encephalitis—therapy is merely supportive for most of them. While steroids have repeatedly been used as adjunctive treatment, evidence for their routine use in VE is scarce. We present a systematic review and meta-analysis on the use of steroids in VE covering all typical viral pathogens as well as a mixed adult and pediatric population.

## Methods

We conducted a systematic review and report it according to the Preferred Reporting Items for Systematic Reviews and Meta-Analyses (PRISMA) standards [3]. Main outcomes assessed were death/survival and number of adverse events.

We performed a MEDLINE literature search using PubMed to identify all reports as of January 31, 2020 using the search terms (“Encephalitis, Viral”[Mesh]) AND “Steroids”[Mesh] and (“Encephalitis, Viral”[Mesh)] AND “Glucocorticoids”[Mesh]. Other databases searched included the Cochrane Database, Biosis Previews, and the ClinicalTrials.gov website. Titles and abstracts of the reports were screened for inclusion in the review using the following criteria: population with VE (polyomavirus infections, prion diseases, and subacute sclerosing panencephalitis were excluded); at least part of the population treated with steroids; outcome and/or safety of steroid therapy reported. Reports on patients with steroid therapy for pre-existing conditions or use of steroids explicitly for treatment of cerebral edema were excluded. Exclusion criteria were based on the intention to increase the homogeneity of the population under investigation.

Articles published in languages other than English, German, or Spanish as well as preclinical studies, editorials and reviews (except for secondary search) were excluded. Included were all case reports, case series (five or more patients), retrospective and prospective observational studies, and randomized controlled trials. Secondary search for other relevant articles was performed in the articles included after full-text analysis as well as in reviews on the topic. The searches and data extraction were performed by JW. The final decision as to the inclusion of a report lay with JW.

The following data were extracted: study type and design, patient demographics, number of patients, presence vs. absence of immunosuppression, causative pathogen, Glasgow Coma Scale score at admission, need for intensive care unit admission (ICU admission was assumed if either explicitly mentioned or procedures requiring intensive care were performed), initial CSF cell count, timing, duration, and dosing of steroids and specific steroid used, route of application, concomitant therapies relevant to viral infections, outcome (complete remission/improvement/no improvement/death/survival, no further outcome data) and estimated modified Rankin Scale score at discharge and last follow-up if available, and adverse events. Steroid dosages were normalized to the equivalent dexamethasone dose. For “herpes simplex virus (HSV)” without specification of the subtype, HSV1 was assumed. Main outcomes assessed were survival for observational studies and clinical trials at last patient contact after the acute disease (day of hospital discharge for most patients; day of follow-up if no prior data available). Secondary outcome parameters are listed in the results section if available from the reports. The safety outcome was defined as number of severe adverse events.

Two more patient cohorts from Linz (Austria) and Houston (Texas, USA) were added. Data of all patients with the International Classification of Diseases ICD-9 and ICD-10 discharge diagnosis of encephalitis who were treated between 2007 and 2017 at one of the two neurological departments of the Kepler University Hospital Linz, Austria, or between 2008 and 2017 at two tertiary care hospital systems in Houston, Texas (15 hospitals from the Memorial Hermann Health System and two hospitals from the Harris Health System) were reviewed. Those patients with a diagnosis of encephalitis according to the 2013 International Encephalitis Consortium definition with a confirmed viral pathogen or with a high likelihood of a viral etiology (monophasic disease, mild to moderate pleocytosis on first cerebral spinal fluid (CSF) analysis) were included [1]. Clinical data were retrieved from the electronic patient data file. The data of the Houston cohort have partially been published elsewhere [4].

Statistical analysis was performed by EH and HW using the statistical software R-4.3.1. A systematic assessment of the available evidence was conducted using the GRADE (Grading of Recommendations Assessment, Development and Evaluation) methodology [5, 6]. Publication bias was investigated via a funnel plot. For studies with group data, we contacted the corresponding authors to obtain individual data. Data were summarized using descriptive statistics. The association of steroid therapy and survival was investigated using odds ratios and Fisher’s exact test. For the meta-analysis, we employed the metaphor package for R and fitted a random effects model. The random effects model was chosen as the different studies with widely differing time points suggest substantial heterogeneity across the studies. Due to the paucity of clinical outcome data and the therapeutic heterogeneity, we only investigated the effect of any steroid treatment on survival of the patient. For mortality, we calculated the odds ratio (OR) with 95% confidence intervals. We hypothesized that the OR for survival with and without steroid treatment equals 1 (null hypothesis; OR = 1). We tested the studies for heterogeneity using the *I*^2^ statistic as a measure of inconsistency.

Standard Protocol Approvals, Registrations, and Patient Consents: The Houston study was approved by the University of Texas Health McGovern Medical School Institutional Review Board. The remaining study was exempt from ethical approval procedures by the Ethics Committee of Upper Austria. There was no funding source for the study. The corresponding author had full access to all the data in the study and had final responsibility for the decision to submit for publication. The review was registered with the PROSPERO database (registration number CRD42021233965).

Data availability: Data not provided in the article because of space limitations may be shared (anonymized) at the request of any qualified investigator for purposes of replicating procedures and results.

## Results

**Fig. 1:**
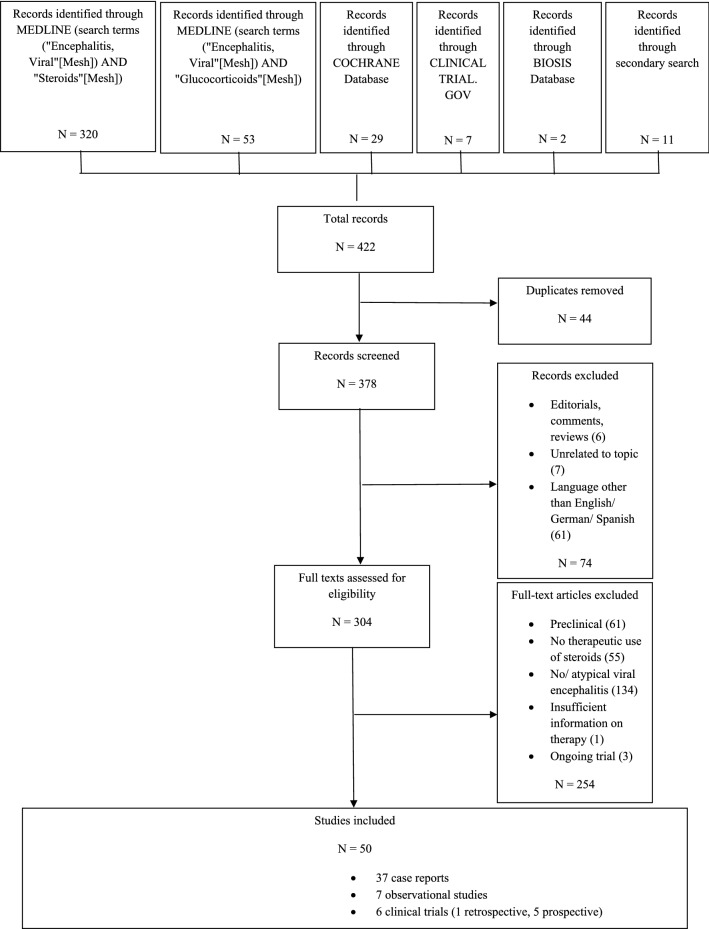
Selection of included reports. Flow chart depicting the selection process of reports included in this review according to PRISMA guideline

We screened a total of 378 studies, 50 of which were included (see Fig. 1): five prospective trials, one retrospective trial, seven observational studies, and 37 case reports. Two observational studies from unpublished or partially published data were added (Linz/Houston cohort).

### Case reports

The case reports cover a total of 43 patients (20 adults, 23 children; for details see Table S1 (supplementary materials) and Table 1), 33 from single case reports, 10 from case series covering four patients or less.

**Table 1:** Details case reports

		Children	Adults
Pathogen; *n*			
Herpes simplex virus	14	6	8
Influenza A	4	2	2
Epstein–Barr virus	4	2	2
Parvovirus B19	4	4	0
Varicella zoster virus	4	4	0
Intensive care treatment; *n*	12		
Median CSF cell count (range); cells/µl	50 (0–1196)		
Therapy as add-on to steroids; *n*			
Acyclovir	24		
i.v. immunoglobulins	11		
(Val)ganciclovir	2		
Plasmapheresis	2		
Others	10		
Steroid monotherapy	8		
Steroid compounds used; *n*			
(Methyl)prednisolone	20		
Dexamethasone	20		
Hydrocortisone	0		
Not specified	3		
Modified ranking scale score discharge; *n*			
0	6		
1	2		
2	2		
3	5		
4	3		
5	1		
6	4		
Modified ranking scale score follow-up			
0	8		
1	2		
2	5		
3	1		
4	5		
5	3		
6	0		

Frequency of pathogens (listed if > 2 cases), frequency of ICU admission, and details on therapies administered. Modified rankin scale scores at discharge and last follow-up

Modalities of steroid administration varied widely. The median daily dose (dexamethasone equivalent) was 40 mg (range 8–188 mg; 22 patients). For 16 patients, the daily dose was available relative to body weight (median 2 mg/kg; range 0.1–16.9 mg/kg). For 18 patients, enough information was available to calculate the total steroid dose they received. The median total dose was 180 mg (range 24–940 mg; dexamethasone equivalent). The median start of steroid therapy was seven days after symptom onset (specified for 20 patients; range 1–26) and 3.5 days after hospital admission (specified for eight patients; range 1–7). The median duration of steroid application was four days (28 patients; range 2–25 days). For other minutiae of dosing, duration, and route of administration, see Tables S1 and 1. The authors of only two cases reported by the same group explicitly mention adverse effects (hypernatremia, hyperglycemia, and hypokalemia) [7]. On final evaluation, 12 patients had completely recovered, 22 improved, five were alive without improvement and four had died. Rankin Scale scores were available or could be estimated from the clinical data for 23 patients at discharge and for 24 patients at follow-up (median 240 days; range 30–1095 days; see Table 1).

### Studies and case series

Details and GRADE ratings of the published trials and case series on the use of steroids in VE are specified in Table 2. Three of the trials (2 prospective, 1 retrospective) report an advantage for steroid therapy.

**Table 2:** GRADE assessment of the included trials and observational studies

Author	No. of patients	Details on steroid therapy Preparations used Duration Time point of initiation	Population	Date of recruitment	Pathogen	Design	Outcome	Quality of evidence
Allen* JE [16]	10	Cortisone acetate 525 mg total 3 days Within 24hs of admission	Pediatric	07/1957	Measles	Observational study	Survival Clinical outcome Adverse effects	Very low^b, c, d, e, g^
Chaari* et al. [18]	21	Dexamethasone 0.6 mg/kg/day 2–5 days No data available	Pediatric	2011	Rubella	Observational study	Survival Clinical outcome	Very low^b, d, e, f, g^
Duniewicz et al. [9]	138 (hydrocortisone i.v.—35; hydrocortisone i.m.—37; methylprednisolone—26; prednisone—11; controls—29)	Hydrocortisone max. 10 mg/kg/day (taper); methylprednisolone max. 2.5 mg/kg/day; prednisone 1 mg/kg/day 3–6 days No data available	No information	?	Miscellaneous (presumed viral)	Prospective trial	Survival Clinical outcome	Very low^b, d, e, f, h, i^
Hoke et al. [15]	55 (25 dexamethasone, 30 placebo)	Dexamethasone 0.8 mg/kg 5 days No data available	Adult/pediatric	06-08/1984	JEV	Prospective trial	Survival Clinical outcome Adverse effects Laboratory parameters	Low^a, B^
Johnson* et al. [14]	13 (6 steroids, 7 no steroids)	Dexamethasone 0.8 mg/kg 5 days No data available	Adult/pediatric	06-08/1984	JEV	Prospective trial	Survival Clinical outcome CSF: cellular composition	Very low^b, d, e, f, i^
Kamei* et al. [10]	45 (22 dexamethasone or prednisolone; 23 controls)	Prednisolone, dexamethasone 40–96 mg/day (prednisolone equivalent) 2–42 days 3–12 days after onset	Adult	1996–2004	HSV-1	Retrospective trial	Survival Clinical outcome	Very low^b, e, f, g, i^
Karelitz* et al. [12]	20 (14 steroids, 6 no steroids)	Cortisone (525–600 mg total); hydrocortisone (25–500 mg total); methylprednisolone (200–800 mg total) 2–5 days Within 24 h of admission	Pediatric	1952–1959	Measles	Observational study	Survival Clinical outcome	Very low^b, c, e, f, g, i, j^
Koskiniemi* et al. [17]	7 (3 proven HSVE, combination interferon/vidarabine/dexamethasone; 3 presumed HSVE, combination interferon/vidarabine/dexamethasone; 1 presumed HSVE, combination interferon/ dexamethasone)	Dexamethasone 20 mg/d (taper) 10 days 4–1060 days after onset	Adult	?	HSV	Observational study	Survival Clinical outcome Adverse effects	Very low^b, c, d, e, f^
Nakano* et al. [19]	5	Methylprednisolone 1000 mg/day 3 days 4–43 days after onset	Adult	1998–2001	2xJEV, 2xHSV, 1xND	Observational study	Survival Clinical outcome Adverse effects	Very low^b, c, d, e, f, g^
Rathi et al. [13]	91 (35 prednisolone, 56 no steroids)	Prednisolone 1 mg/kg/day No data available 21 days after onset	Pediatric	09–11/1988	JEV	Prospective trial	Survival Clinical outcome	Very low^f, g, j^
Sarkari et al. [8]	1199 (737 dexamethasone; 462 controls)	Dexamethasone 12 mg/day 7 days no data available	Adult (> 14 years)	1978–1989	JEV	Prospective trial	Survival Clinical outcome Adverse effects	Low^a, C, E^
Sauer* et al. [20]	6 (1 dexamethasone, 2 unspecified steroids, 3 no steroids)	Dexamethasone 16 mg/day 4 days within 72hs of admission	Adults	1975	SLEV	Observational study	Survival Clinical outcome	Very low^b, c, e, f, g,, j^
Zhang et al. [11]	80 (40 methylprednisolone; 40 controls)	Methylprednisolone 10 mg/kg/day 3 days no data available	Pediatric	03–09/2014	EV	Prospective trial	Survival Clinical outcome Adverse effect	Low^b, E, F^

*Individual data available

*n.c.* not calculable, *n.a.* not applicable, *HSV* herpes simplex virus, HSVE herpes simplex virus encephalitis, *EV* enterovirus, *JEV* Japanese encephalitis virus, *SLEV* St Louis encephalitis virus, *IVIG* intravenous immunoglobulins, > changed to, *i.v.* intravenously, *i.m.* intramuscular

^a^Unclear risk of detection bias

^b^Small sample size

^c^No control group and/or no randomized design

^d^Heterogeneous cohort

^e^High risk of selection and allocation bias

^f^High risk of detection bias

^g^Retrospective design

^h^Lack of information of part of cohort

^i^Lack of information on treatment protocol and/or heterogeneous treatment

^j^Loss to follow-up

Sarkari et al. treated 737 Japanese Encephalitis (JE) patients recruited during epidemics between 1978 and 1989 with a 7-day course of dexamethasone (12 mg/day) and compared the clinical outcome to 462 controls [8]. Only after exclusion of pulmonary edema cases, who were transferred to the steroid group as a life-saving measure, do they report a significantly lower mortality (42.86% and 36.72%, *p* < 0.01) in the treatment group. No differences concerning adverse events are reported.

Duniewicz et al. found a more rapid normalization of elevated temperatures, headache, nausea, and dizziness in 35 patients with encephalitis caused by diverse viral pathogens treated with intravenous hydrocortisone (5–10 mg/kg/d) than in a control group (29 patients) [9]. The numbers of deceased patients are not explicitly stated; neither is the incidence of adverse event.

Kamei et al. report a more favorable clinical outcome in 22 HSV-1 encephalitis patients treated with dexamethasone or prednisolone at 40–96 mg prednisolone-equivalent per day for a heterogeneous period of time (2–42 days) [10]. The OR for complete recovery is 1.62 (95% confidence interval CI 0.45–5.78). More patients died in the treatment group. However, this odds ratio (OR) did not reach significance. Furthermore, an allocation bias is likely in this non-randomized retrospective trial. No data on adverse events is available.

The other trials report no benefit of steroids in the intervention groups:

Zhang et al. did not see a significant difference in survival between the treatment and control group. The OR for complete recovery is not significant at 0.73 (95% CI 0.15–3.49). The authors describe a significantly higher heart rate, respiratory rate, white blood cell counts, and blood glucose in the patients treated with steroids [11].

Karelitz et al. reported no deaths in their cohort of children with encephalitis [12]. A total of 9 out of 14 treated with steroids and 2 out of 6 non-treated children were asymptomatic at discharge (OR 3.6; 95% CI 0.48–27.11). Adverse effects were not reported.

Rathi et al. describe a trend toward higher mortality in the steroid group [13]. An allocation bias is likely as steroid treatment was not randomized and only applied in those without clinical recovery at three weeks; hence, steroid-treated patients were presumably more affected. The OR for complete recovery was 0.63 (95% CI 0.26–1.51). No data are given for adverse events.

There may be an overlap in the patient cohorts reported by Johnson et al. and Hoke and al, as these were recruited in the same time period in the same hospital [14, 15]. Survival did not differ significantly in the treatment and non-treatment groups. Neither of the two groups describe relevant differences in the occurrence of adverse events.

For the Houston and Linz cohorts (details see Table 3), we found a non-significant tendency toward higher mortality in the treatment group (OR 4.27; 95% CI 0.98–18.69 and OR 6.25; 95% CI 0.22–190.85). As these are retrospective data, we assume that this trend is due to an allocation bias of more severely diseased patients to the treatment group.

**Table 3:** Details of the Houston and Linz cohort

	Houston cohort (Hasbun and Granillo)	Linz cohort (Wagner et al.)
Total number of patients; *n*	83	72
Immunosuppressed; *n*	19	7
Pathogen; *n*		
Cytomegalovirus	4	1
Enterovirus	2	0
Epstein–Barr virus	4	4
Herpes simplex virus	19	12
Influenza A virus	0	1
Parvovirus B19	0	0
St. Louis Encephalitis virus	4	0
Tick-borne Encephalitis virus	0	27
Unspecified	0	22
Varicella zoster virus	17	6
West Nile virus	33	0
Intensive care treatment; *n*	33	26
Median CSF cell count (range); cells/µl	75 (0–1100)	80.5 (2–840)
Median admission GCS (range); score	14 (3–15)	n.a
Steroid therapy; patients; *n*	28	3
Antiviral therapy; patients; *n*	69	65
Median modified Ranking scale score at last contact (range); score	3 (0–6)	1 (0–6)
Patients dead at last contact; *n*	9	1

The outcome of the case series was heterogeneous: full recovery in 10 children with measles encephalitis [16], death or severe sequelae in 4 out of 7 patients with HSV encephalitis in the pre-acyclovir era [17], two fatalities and 19 full recoveries in 21 patients with rubella encephalitis [18], and full recovery in five adults with encephalitis due to diverse pathogens (JE, HSV, and unknown) [19]. All patients received steroid treatment. Sauer described six patients with St. Louis Encephalitis virus, three of which received steroid treatment [20]. One of the non-treated patients died, one of the treated patients recovered fully. The remainder improved with sequelae. Only two groups reported steroid-associated adverse events [17, 19].

### Analysis of individual study data

**Fig. 2:**
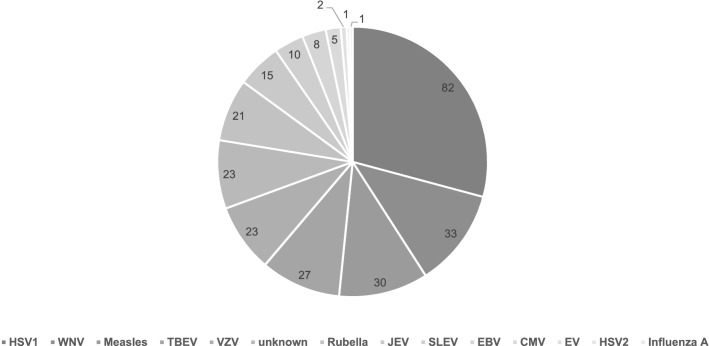
Pathogens in the patient cohort extracted from studies providing individual data

Including the Linz and Houston cohorts, we reviewed 15 studies covering 1836 patients. Group data were available for 1555 patients covered in five studies. Individual data were available for 281 persons (* in Table 2). 26 were immunosuppressed (no data for 118 patients). For causative pathogens, see Fig. 2. Data on the CSF cell count were available for 211 patients. The median cell count was 72 cells/µl (range 0–1376 cells/µl). The median GCS score at admission was 12 (*n* = 129; range 3–15). 82 out of 183 patients received intensive care treatment (missing data *n* = 98).

A total of 120 (43%) out of 279 patients received steroids (missing data: *n* = 2). The substances most commonly used were dexamethasone (*n* = 69), cortisone (*n* = 16), and methylprednisolone (*n* = 15). Route of application was intravenously in 37 patients, intramuscular in 16, and orally in eight patients (missing data: *n* = 220). 162 patients had one, ten patients two, and 26 patients no further antiviral therapy (missing data: *n* = 83). The most frequently used antiviral therapy was acyclovir (*n* = 135), other substances included ACTH, interferons, intravenous immunoglobulins, (val-)ganciclovir, and vidabirine.

The median daily steroid dose (dexamethasone equivalent) was 16 mg (range 3–188 mg; 51 patients) and 0.6 mg/kg (range 0.6–0.8 mg/kg; 27 patients). The median total dose was 44 mg (range 10–1880 mg; 43 patients) and 1.8 mg/kg (range 1.8–4.6 mg/kg; 27 patients). Steroid therapy was started at a median of five days after symptom onset (range 1–1060; 53 patients). The median duration of steroid application was four days (range 1–30 days; 65 patients). Side effects were reported for six patients (hyperglycemia, leukopenia, elevated liver function parameters, polyuria, and thrombopenia; missing data: *n* = 190).

On final evaluation, 106 patients had completely recovered, 75 improved, eight were alive without improvement and 26 had died. 61 patients survived; however, no further data as to their clinical outcome were available. Outcome data were missing for five patients. The time from onset to final evaluation was available for 72 patients: median 19 days, range 4–116 days.

127 patients had a follow-up with a median of 180 days (last follow-up; range 44 days– > 13 years). 13 additional patients had died in the meantime. 66 patients showed no and 17 patients minor symptoms (modified Rankin Scale (mRS) score 0 or 1), 54 patients had a mRS score between 2 and 5 (missing data *n* = 131).

**Fig. 3:**
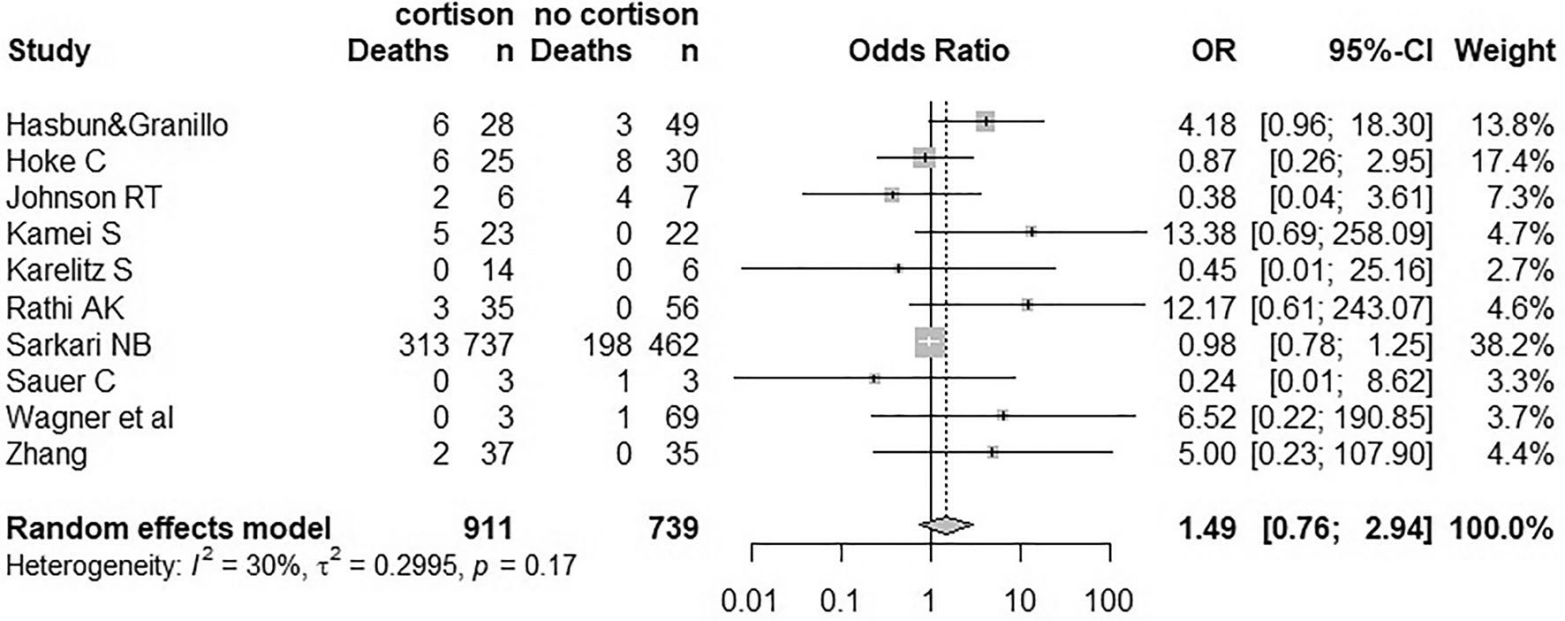
Forest Plot of the odds ratio (OR) for the risk of dying in patients who received steroids (C.P.) vs. those who did not (no C.P.)

For the meta-analysis, data by Hasbun et al., Hoke et al., Johnson et al., Kamei et al., Karelitz et al., Rathi et al., Sarkari et al., Sauer et al., Wagner et al., and Zhang et al. were included [8, 10–15, 20]. Visual inspection of the funnel plot suggests presence of a publication bias. However, only few studies have been included into the meta-analysis. With *I*^2^ = 30.2%, heterogeneity across studies seems low. For the pooled data, the null hypothesis could not be rejected (*p* = 0.248) using a random effects model, i.e., a beneficial or harmful effect of steroid treatment on survival in VE could not be shown (for Forest Plot see Fig. 3). The same is true for all individual virus subgroups investigated (HSV, West Nile virus, varicella zoster virus, JEV, Saint Louis encephalitis virus, Epstein–Barr virus, and cytomegalovirus).

## Discussion

Extensive intracerebral inflammation in VE has been described in animal models as well as in human patients [21, 22]. Significant neurological damage may be due by an unchecked reaction of the host’s immune system—so-called cytokine storm—rather than by viral invasion per se. This has, among others, been reported for pathogens, such as enterovirus, influenza virus, and the novel coronavirus SARS-CoV-2 [23–25]. Steroids are potent anti-inflammatory agents and may act through a reduction of secondary inflammation-mediated damage. Their benefits have been shown for other types of meningoencephalitis, such as pneumococcal meningitis or tuberculosis [26].

Although studies detailing VE patients in whom steroids were applied as a life-saving measure in case of increased intracerebral pressure and reports on patients with post-viral autoimmune disease were excluded from this analysis, further beneficial effects of steroid therapy in VE do include anti-edematous action and possible prevention or therapy of secondary autoimmune phenomena. HSV encephalitis, for instance, is typically accompanied by extensive cerebral edema, which is prone to increase morbidity and mortality in affected patients. Furthermore, herpes viruses are associated with a range of secondary autoimmune diseases such as vasculitis or encephalitis associated with anti-neuronal antibodies [27–29]. Steroid effects on these pathologies may help to explain the differential benefit in various types of VE.

Results from animal studies as to the effect of steroids on viral disease have been heterogeneous. While some authors describe an exacerbation or unchanged outcome in steroid-treated animals [30], others see more favorable results [31, 32]. In a mouse model of HSV-1 encephalitis, a reduction of chronic progressive magnetic resonance imaging (MRI) changes was detected in mice treated with methylprednisolone and acyclovir compared to those on acyclovir monotherapy [33]. Viral load did not differ between the two groups, suggesting that steroids do not promote virus replication. Interleukin 6 (IL-6) has been shown to be an important factor in the inflammatory cascade following HSV-1 infection [34, 35]. High CSF-levels of IL-6 have been found to herald a poor prognosis in HSV-1 encephalitis. Steroids reduce IL-6 and other inflammatory cytokines [36]. On the other hand, reservations about the use of steroids in VE have been voiced fearing a possible exacerbation of viral replication due to their immunosuppressive effect [37].

Evidence that timing of steroid application may be crucial has been obtained in cell cultures and animal models [38, 39]. In mice infected with HSV-1, mean life expectancy was higher in mice receiving a delayed (i.e., 72 h post-inoculation) steroid treatment compared to those immediately treated with steroids and to controls. The authors conclude that the acute-phase reaction may be vital to efficient virus control, whereas secondary, probably NF-kB-associated signaling pathways, may lead to neuro-destructive inflammation. Pre-clinical and clinical data on HSV-1 encephalitis are assembled in a systematic review, which delineates the temporal course of this disease [40]. The authors conclude that delayed adjunctive steroid therapy may be recommended.

Although pathophysiological aspects are similar for many viral infections, it remains open if HSV-specific recommendations can be extended to other viruses. For example, the effect of steroids may depend on the main route of viral spread into the central nervous system. HSV is propagated inter-neuronally. Different patho-mechanisms may be relevant for hematogenic infections such as JE. In an in vitro model of the human blood–brain barrier (BBB), JEV infection led to increased cytokine production by epithelial cells, causing disruption of the BBB and subsequent viral transmission [41]. Dexamethasone reduced cytokine production and BBB permeability without enhancing JEV replication. However, a descriptive study on West Nile virus disease did not report an advantage of steroid treatment of those patients classified as VE [42].

The data included in this review and meta-analysis do not yield superiority of steroid therapy compared to supportive treatment or antiviral monotherapy, nor does it favor a specific steroid compound, timing or dosing regimen. Therapeutic approaches in the studies investigated vary considerably and seem to be informed by local conventions rather than by pathophysiological rationales (see Table 2). To some extent, this may explain the higher rate of steroid use in the Houston versus the Linz cohort, as this discrepancy extends to all pathogens occurring at both sites. Another factor might be the differences in morbidity and mortality, pointing toward a more severely affected cohort treated at the Houston site.

In the three trials reporting a positive outcome, most patients were treated with dexamethasone at equivalent doses of five to 30 mg per day, well below the dose of 40 mg per day recommended in adults with bacterial meningitis [8–10, 43]. Most patients in these trials received steroids for 7 days or less, presumably owing to the idea that the hyper-acute phase of VE has passed after this period and potential steroid side effects will surpass its benefit. Only one of the positive trials reports the interval between VE onset and steroid initiation (3–12 days); hence, no valid conclusions can be drawn [10].

The data generated by the case series and clinical trials are of low to very low quality due to small and heterogeneous study populations and interventions, incomplete data, and possible selection, allocation and detection bias. The generalizability of the results to other pathogens and different socioeconomic settings is questionable. Furthermore, although cases reporting the use of steroids explicitly for increased intracerebral pressure were not considered, we cannot exclude that beneficial effects of steroids were mediated via the anti-edematous rather than the antiviral or anti-inflammatory effect.

Steroid use seems to be safe in most cases. Only one trial claims that side effects were more frequent in the steroid than in the control group [11]. It does not become evident if this affected the clinical outcome in the treatment group. Furthermore, the trial pertains to an extremely ill cohort receiving multiple drugs. Thus, it remains open if the side effects were steroid-induced [17]. No or only minor transient adverse effects were reported in the other case reports and case series. However, publication bias is likely. Due to the lack of a randomized design in most trials and the paucity of specific outcome data, we cannot fully exclude that steroid use leads to a worse outcome as defined by the mRS. However, our meta-analysis did not support a harmful effect of steroids concerning survival.

In total, a general recommendation as to the routine use of steroids in VE cannot be made at this point. This does not apply to other indications, such as cerebral edema or para-/post-infectious autoimmune complications. Concordantly, recommendations for the use of steroids in VE rest on class IV evidence [44]. If a specific antiviral treatment exists—such as acyclovir in herpes simplex encephalitis—the priority is to avoid delay in starting this therapy. However, our data show that non-herpes viruses are common in most cohorts of VE patients and that the clinical outcome is unfavorable for many of them, calling for clinical trials on alternative therapeutic options.

One clinical trial on the use of steroids in herpes simplex virus encephalitis (GACHE trial, ISRCTN45122933) was terminated prematurely due to slow recruitment [45, 46]. With 21 patients enrolled in the steroid and 20 patients in the placebo arm, no significant differences were seen for the primary endpoint (score on the mRS after 6 months) or for any other outcome measure. Encouragingly, no serious adverse effects were described in the patients treated with dexamethasone. Two more trials are on the way: one investigating the effect of low- vs. high-dose steroids in in children with VE (NCT04103684), another on the benefit of dexamethasone in herpes simplex virus encephalitis in adults (DexEnceph; NCT03084783).

Our analysis demonstrates that treatment protocols are extremely heterogeneous concerning steroid compound, dosing, duration of therapy and route of administration. Thus, trials should not only investigate the efficiency of steroids in different viral pathogens per se, but also focus on the optimal dosing and timing of steroid administration. The latter may be further optimized by assessing serum and CSF markers of inflammation and neuronal destruction. MRI surrogates may be helpful in those viral pathogens such as HSV typically leading to abnormalities on cerebral imaging.

## Supplementary Information

Below is the link to the electronic supplementary material.

Supplementary file1 (DOCX 57 KB)

## Data Availability

All data are available from JNW upon request.
