# Supplementary material for: Steroids for the treatment of viral encephalitis: a systematic literature review and meta-analysis
Source: J Neurol. 2023 Apr 15;270(7):3603–15. doi: 10.1007/s00415-023-11715-0 (PMC10105360; doi:10.1007/s00415-023-11715-0)
Supplement: Supplementary file 1 — Supplementary file1 (DOCX 57 KB) [file 415_2023_11715_MOESM1_ESM.docx]

| **Autor** | **Children (n)** | **Adults (n)** | **Patho-gen** | **Substance** | **Route of administration** | **Max. dose per day** | **Day of therapy start (from hospitalization/ symptom onset)** | **Total duration of theray (days)** | **Mono- vs. add-on therapy*** | **Outcome at discharge/ last patient contact** |
| --- | --- | --- | --- | --- | --- | --- | --- | --- | --- | --- |
| **Almalki et al.**[1] | 0 | 1 | HSV-1 | methylprednisolon | iv | 500mg | ? | 5 | add-on (acyclovir) | clinical improvement |
| **Dale et al.**[2] | 1 | 0 | VZV | methylprednisolone -> prednisolone | iv -> po | ? | ? / 5 | 47 | add-on (acyclovir) | clinical improvement, resolution of MR lesion |
| **Enoki et al.**[3] | 1 | 0 | HHV-6 | dexamethasone -> methylprednisolone | ? | 0,8mg/kg -> ? | ? /2 | 7 | mono | clinical improvement |
| **Erol et al.**[4] | 1 | 0 | hPV-B19 | methylprednisolone -> prednisone | iv | 20mg/kg -> ? | ? / 20 | 3 -> 60 | add-on (acyclovir, IVIG) | clinical improvement (seizures) |
| **Fay et al.**[5] | 1 | 0 | HHV-7 | steroids | ? | ? | ? / 10 | ? | add-on (acyclovir, IVIG, plasma exchange) | clinical improvement |
| **Fernández-Blázquez et al.**[6] | 0 | 1 | Inf A | dexamethasone | ? | 24mg | ? | ? | Add-on (acyclovir, oseltamivir) | dead |
| **Garzo-Caldas et al.**[7] | 0 | 1 | EV | methylprednisolone | ? | 1000mg | ? | 5 | add-on (IVIG) | dead |
| **Georgescu et al.**[8] | 1 | 0 | EV | dexamethasone, methylprednisolone, hydrocortisone | ? | ? | ? | 10 | add-on (IVIG, acyclovir) | Psychomotor retardation |
| **Golomb et al.**[9] | 0 | 1 | EEE | methylprednisolone | iv | 1000mg | ? | ? | add-on (aciclovir, IVIG) | full recovery |
| **Greco et al.**[10] | 1 | 0 | hPV-B19 | dexamethasone | iv | 1mg/kg | ? / 3 | ? | add-on (acyclovir, IVIG) | inital clinical improvement, secondary ataxia |
| **Grillo et al.**[11] | 1 | 0 | EBV | dexamethasone -> methylprednisolone | ? | ? | ? | ? | mono | clinical improvement |
| **Häusler et al.**[12] | 2 | 0 | VZV | dexamethasone | ? | 2mg/kg | ? / ?; ? / 10 |  | add-on (acyclovir) | clinical improvement |
| **Hindo et al.**[13] | 1 | 0 | WNV | steroids | iv | ? | 4 / 11 | 3 | add-on (aciclovir, G-CSF, IVIG) | dead |
| **Ito et al.**[14] | 0 | 1 | Inf A | methylprednisolon | ? | 1000mg | ? / 4 | 3 | add-on (oseltamivir) | clinical and radiological improvement |
| **Kimura et al.**[15] | 0 | 1 | NV | methylprednisolon | iv | 1000mg | ? / 5 | 3 | add-on (acyclovir, IVIG) | clinical improvement |
| **Krishnan et al.**[16] | 0 | 1 | HHV-6 | methylprednisolone | ? | 1000mg | ? | 5 | add-on (acyclovir, plasmapheresis) | dead |
| **Lizarraga et al.**[17] | 0 | 1 | HSV-1 | dexamethasone | iv | 40mg | 5 / 26 | 5 | add-on (acyclovir) | clinical improvement |
| **Mesker et al.**[18] | 0 | 1 | HSV-1 | dexamethasone |  | 40mg | ? / 11 | 4 | add-on (acyclovir) | clinical improvement |
| **Munakata et al.**[19] | 2 | 0 | Inf A | methylprednisolone | iv | 90mg/kg | 1 / 2 (2x) | 4; 3 | add-on (amantadine, ulinastatin) | clinical improvement |
| **Musallam et al.**[20] | 1 | 0 | HSV-1 | methylprednisolon | iv | 1000mg | ? | 3 | add-on (acyclovir) | clinical improvement |
| **Navin et al.**[21] | 0 | 1 | HSV-1 | dexamethasone | ? | ? | ? | 14 | add-on (acyclovir) | clinical improvment |
| **Piskin et al.**[22] | 0 | 1 | HSV | dexamethasone | ? | ? | 3 / 5 | 25 | add-on (acyclovir) | rapid improvement |
| **Raman et al.**[23] | 0 | 1 | EBV | dexamethasone | ? | 12mg | ? | ? | add-on (valganciclovir) | clinical and radiological impovement |
| **Rodrigo-Armenteros et al.**[24] | 1 | 0 | EBV | methylprednisolone | iv | 10mg/kg | ? | 3 | add-on (acyclovir) | full recovery |
| **Sountharalingam et al.**[25] | 0 | 1 | JEV | methylprednisolon | iv | 1000mg | ? | 5 | mono | clinical improvement |
| **Spiegel et al.**[26] | 1 | 0 | VZV | methylprednisolon | ? | 1000mg | ? | 5 | add-on (acyclovir) | clinical improvement |
| **Suga et al.**[27] | 1 | 0 | mumps | methylprednisolon -> prednisolon | iv -> po | 30mg/kg -> ? | ? | 3 | add-on (IVIG) | clinical deterioration |
| **Takasawa et al.**[28] | 1 | 0 | hPV-B19 | methylprednisolon | iv | 30mg/kg | ? | 3 | mono | clinical improvement (seizures) |
| **Takebayashi et al.**[29] | 0 | 1 | measles | prednisolone | iv | 80mg | 2 / 12 | 2 | mono | good condition, discharge home |
| **Uchida et al.**[30] | 1 | 0 | hPV-B19 | dexamethasone -> methylprednisolone | iv -> ? | ? -> 30mg/kg | ?; 4 / 7 | ? -> 3 | add-on (acyclovir) | clinical improvement, incomplete recovery |
| **Unal et al.**[31] | 0 | 1 | mumps | prednisolone | po | 1mg/kg | ? | ? | mono | clinical improvement, residual paraparesis due to myelitis |
| **Upton et al.**[32] | 0 | 2 | HSV | dexamethasone | ? | 20mg | ?; 1 / 8 | ? | mono | minimal dysphasia; severe memory deficits |
| **Venturini et al.**[33] | 1 | 0 | HSV-1 | dexamethasone | iv | 0,6mg/kg | ? | ? | add-on (acyclovir) | clinical improvment |
| **Villacé et al.**[34] | 0 | 1 | EBV | dexamethasone | iv | 60mg |  |  | add-on (acyclovir, IVIG) | clinical improvement |
| **Wolman et al.**[35] | 4 | 0 | HSV | dexamethasone | iv, im | 2x 1mg/kg -> 8mg/d; 1x 10mg/kg -> 40mg/d; 1 x 8mg/d | ? / ? (3x); ? / 5 (1x) | ? (1x); 5 (3x) | add-on (cytosine arabinoside) | 2xvegetative state;1x unable to stand, walk, and talk; 1x severely retarded |
| **Ye et al.**[36] | 0 | 1 | HSV-1 | methylprednisolone | iv | 120mg | 2/? | 3 | add-on (acyclovir) | clinical improvement |
| **Zaganas et al.**[37] | 0 | 1 | CMV | steroids | ? | ? | ? | ? | add-on (ganciclovir, IVIG) | clinical improvement |

**Table 1. Case reports (max. 4 homogenous patients) included in the review.** HSV – herpes simplex virus; EV – enterovirus; hPV-B19 – human parvovirus B19; EBV – Ebstein-Barr-virus; JEV – Japanese encephalitis virus; HHV-6 – human herpesvirus-6; HHV-7 – human herpesvirus-7; EEE – eastern equine encephalitis; SLEV - St Louis encephalitis virus; WNV – West Nile virus; Inf A – influenza A; NV – norovirus; VZV – varicella zoster virus; CMV – cytomegaly virus; IVIG – intravenous immunoglobulins; -> changed to; iv – intravenously; po – orally

*only therapeutics relevant to viral infections are listed

**Literature**

1. Almalki DM, Al-Suwaidan FB (2013) Steroid pulse therapy in herpes simplex encephalitis. Neurosci Riyadh Saudi Arab 18:276–277

2. Dale RC, Church AJ, Heyman I (2003) Striatal encephalitis after varicella zoster infection complicated by Tourettism. Mov Disord Off J Mov Disord Soc 18:1554–1556. https://doi.org/10.1002/mds.10610

3. Enoki H, Takeda S, Matsubayashi R, Matsubayashi T (2006) Steroid therapy in an infant with human herpesvirus 6 encephalopathy. Brain Dev 28:597–599. https://doi.org/10.1016/j.braindev.2006.03.005

4. Erol I, Alehan F, Yalçin K (2006) Refractory status epilepticus owing to human parvovirus B19 encephalitis in a child. J Child Neurol 21:820–822. https://doi.org/10.1177/08830738060210092301

5. Fay AJ, Noetzel MJ, Mar SS (2015) Pediatric Hemorrhagic Brainstem Encephalitis Associated With HHV-7 Infection. Pediatr Neurol 53:523–526. https://doi.org/10.1016/j.pediatrneurol.2015.06.016

6. Fernández-Blázquez A, Castañón-Apilánez M, Álvarez-Argüelles ME, et al (2019) Neuroinvasion of influenza A/H3N2: a fatal case in an immunocompetent adult. J Neurovirol 25:275–279. https://doi.org/10.1007/s13365-018-0690-9

7. Garzo-Caldas N, Ruiz-Sainz E, Vila-Bedmar S, et al (2017) Enteroviral T-cell encephalitis related to immunosuppressive therapy including rituximab. Neurology 89:408–409. https://doi.org/10.1212/WNL.0000000000004148

8. Georgescu A, Chiriac C, Tilea B, Kezdi I (2012) Severe enteroviral encephalitis complicated by encephalomalacia--case presentation. Rev Med Chir Soc Med Nat Iasi 116:799–803

9. Golomb MR, Durand ML, Schaefer PW, et al (2001) A case of immunotherapy-responsive eastern equine encephalitis with diffusion-weighted imaging. Neurology 56:420–421. https://doi.org/10.1212/wnl.56.3.420

10. Greco F, Barbagallo ML, Chiodo DC, et al (2008) Severe ataxia as a complication of human parvovirus B19 acute encephalitis in a child. J Child Neurol 23:1078–1080. https://doi.org/10.1177/0883073808315420

11. Grillo E, da Silva RJM, Barbato Filho JH (2008) Epstein-Barr virus acute encephalomyelitis in a 13-year-old boy. Eur J Paediatr Neurol EJPN Off J Eur Paediatr Neurol Soc 12:417–420. https://doi.org/10.1016/j.ejpn.2007.10.016

12. Häusler M, Schaade L, Kemény S, et al (2002) Encephalitis related to primary varicella-zoster virus infection in immunocompetent children. J Neurol Sci 195:111–116. https://doi.org/10.1016/s0022-510x(02)00017-5

13. Hindo H, Buescher ES, Frank LM, et al (2005) West Nile virus infection in a teenage boy with acute lymphocytic leukemia in remission. J Pediatr Hematol Oncol 27:659–662. https://doi.org/10.1097/01.mph.0000188111.04459.6f

14. Ito S, Shima S, Ueda A, et al (2011) Transient splenial lesion of the corpus callosum in H1N1 influenza virus-associated encephalitis/encephalopathy. Intern Med Tokyo Jpn 50:915–918. https://doi.org/10.2169/internalmedicine.50.4147

15. Kimura E, Goto H, Migita A, et al (2010) An adult norovirus-related encephalitis/encephalopathy with mild clinical manifestation. BMJ Case Rep 2010:. https://doi.org/10.1136/bcr.03.2010.2784

16. Krishnan P, Ramadas P, Chakravarty R, Sah B (2017) A Rare and Fatal Case of Viral Encephalitis in an Immunocompetent Host. Am J Med Sci 353:194–195. https://doi.org/10.1016/j.amjms.2016.07.006

17. Lizarraga KJ, Alexandre LC, Ramos-Estebanez C, Merenda A (2013) Are steroids a beneficial adjunctive therapy in the immunosuppressed patient with herpes simplex virus encephalitis? Case Rep Neurol 5:52–55. https://doi.org/10.1159/000350572

18. Mesker AJ, Bon GG, de Gans J, de Kruijk JR (2011) Case report: a pregnant woman with herpes simplex encephalitis successfully treated with dexamethasone. Eur J Obstet Gynecol Reprod Biol 154:231–232. https://doi.org/10.1016/j.ejogrb.2010.10.014

19. Munakata M, Kato R, Yokoyama H, et al (2000) Combined therapy with hypothermia and anticytokine agents in influenza A encephalopathy. Brain Dev 22:373–377. https://doi.org/10.1016/s0387-7604(00)00169-8

20. Musallam B, Matoth I, Wolf DG, et al (2007) Steroids for deteriorating herpes simplex virus encephalitis. Pediatr Neurol 37:229–232. https://doi.org/10.1016/j.pediatrneurol.2007.05.007

21. Navin P, Delanty N, Brennan P, Looby S (2013) Herpes simplex virus encephalitis involving the right thalamus. BMJ Case Rep 2013:. https://doi.org/10.1136/bcr-2013-010206

22. Piskin N, Akduman D, Aydemir H, et al (2008) Herpes simplex virus encephalitis in pregnancy. J Matern-Fetal Neonatal Med Off J Eur Assoc Perinat Med Fed Asia Ocean Perinat Soc Int Soc Perinat Obstet 21:421–423. https://doi.org/10.1080/14767050802037993

23. Raman L, Nelson M (2014) Cerebral vasculitis and encephalitis due to Epstein-Barr virus in a patient with newly diagnosed HIV infection. J Clin Virol Off Publ Pan Am Soc Clin Virol 59:264–267. https://doi.org/10.1016/j.jcv.2014.01.018

24. Rodrigo-Armenteros P, Kapetanovic-García S, Antón-Méndez L, et al (2019) Akinetic mutism and status epilepticus due to Epstein Barr virus encephalitis. Clin Neurol Neurosurg 185:105492. https://doi.org/10.1016/j.clineuro.2019.105492

25. Sountharalingam S, Herath HMMTB, Wijegunasinghe D, Senanayke S (2017) Opsoclonus myoclonus syndrome in a patient with Japanese encephalitis: a case report. J Med Case Reports 11:294. https://doi.org/10.1186/s13256-017-1454-5

26. Spiegel R, Miron D, Lumelsky D, Horovitz Y (2010) Severe meningoencephalitis due to late reactivation of Varicella-Zoster virus in an immunocompetent child. J Child Neurol 25:87–90. https://doi.org/10.1177/0883073809336296

27. Suga K, Goji A, Shono M, et al (2015) Mumps encephalitis with akinesia and mutism. Pediatr Int Off J Jpn Pediatr Soc 57:721–724. https://doi.org/10.1111/ped.12581

28. Takasawa K, Takeda S, Nishioka M, et al (2016) Steroid-responsive Status Epilepticus Caused by Human Parvovirus B19 Encephalitis. Pediatr Infect Dis J 35:227–228. https://doi.org/10.1097/INF.0000000000000979

29. Takebayashi K, Aso Y, Wakabayashi S, et al (2004) Measles encephalitis and acute pancreatitis in a young adult. Am J Med Sci 327:299–303. https://doi.org/10.1097/00000441-200405000-00034

30. Uchida Y, Matsubara K, Morio T, et al (2012) Acute cerebellitis and concurrent encephalitis associated with parvovirus B19 infection. Pediatr Infect Dis J 31:427. https://doi.org/10.1097/INF.0b013e3182481e24

31. Unal A, Emre U, Atasoy HT, et al (2005) Encephalomyelitis following mumps. Spinal Cord 43:441–444. https://doi.org/10.1038/sj.sc.3101735

32. Upton AR, Barwick DD, Foster JB (1971) Dexamethasone treatment in herpes-simplex encephalitis. Lancet Lond Engl 1:290–291. https://doi.org/10.1016/s0140-6736(71)91019-1

33. Venturini E, Chiappini E, Fonda C, et al (2013) Herpes simplex encephalitis with occipital localization in an infant: a different route of entry in the brain system? Pediatr Neurol 48:463–465. https://doi.org/10.1016/j.pediatrneurol.2013.02.013

34. Villacé P, Navarro M, Font B, Segura F (2008) [Acute cerebellar ataxia complicating infectious mononucleosis]. Enferm Infecc Microbiol Clin 26:672–673. https://doi.org/10.1016/s0213-005x(08)75287-x

35. Wolman B, Longson M (1977) Herpes encephalitis. Acta Paediatr Scand 66:243–246. https://doi.org/10.1111/j.1651-2227.1977.tb07842.x

36. Ye L, Ding X, Shen S, et al (2019) Fulminant bilateral acute retinal necrosis complicated with secondary herpes simplex type-1 viral encephalitis: A case report. Medicine (Baltimore) 98:e17001. https://doi.org/10.1097/MD.0000000000017001

37. Zaganas I, Prinianakis G, Xirouchaki N, Mavridis M (2007) Opsoclonus-myoclonus syndrome associated with cytomegalovirus encephalitis. Neurology 68:1636. https://doi.org/10.1212/01.wnl.0000262766.50747.27
